# Role of Cultural Resources in Mental Health: An Existential Perspective

**DOI:** 10.3389/fpsyg.2022.860560

**Published:** 2022-04-18

**Authors:** Shashwat Shukla

**Affiliations:** Department of Management, University of Allahabad, Allahabad, India

**Keywords:** mental health, festivals, existential psychotherapy, yoga, ayurveda, Vedanta

## Abstract

A reductionist view of mental health tends to give limited results. While some important benefits are still achieved, other key elements are left unaddressed. These gaps tend to wipe out the gains which were made by focusing on the dominant aspects of mental health that are promoted by a reductionist view. This paper explores such gaps by looking at those healing traditions which view health and wellness from a broader perspective. Through the live experience of such traditions the paper tries to illustrate how the deeper aspects of mental health are also relevant. The paper attempts to argue that diverse cultures have inbuilt repositories of existential wisdom which can help in promoting and maintaining positive mental health through a conceptual exploratory analysis.

## Introduction

What role does culture play in mental health? The question has been explored in various ways and this article looks at it from an existential, dialogical and ontological perspective. The question assumes significance because the role of self is important in mental health (van Deurzen, 2012). This is so because the lived experience of an individual may become structured, overbearing, rigid and accessing deeper levels of self can help individuals in many ways. It enables them to create spaces for mental health and rejuvenation (Schneider, 2009). How can this rejuvenating aspect of self be used for mental health? This article explores this question in the phenomenological settings of Indic festivals and their spiritual moorings. In this process the article argues toward the need for a broader discourse about mental health.

To enliven agency wherein individuals can use their ontological resources for mental health requires a cogent sense of self. Such a self can help the individual in many ways such as by creating a sense of direction and reducing dysfunctional ambivalence. Yet in a world of competing narratives and shared meanings, finding an enduring, lasting sense of self is a difficult proposition. Few people are able to attain a selfhood which can come in contact with the world and not get disturbed by it. The role of culture in attaining such selfhood is intriguing. The more vocal view seems to be that culture can act as a barrier in the process of knowing oneself. At the same time, it has also been argued that self is itself a social construct. Whatever view one may hold, it is evident that the role of culture vis-a-vis self needs to be given importance. Therefore culture in a regressive form has come to be associated with dogma, superstition and rituals which hamper the mind in forming cogent and correct opinions about self or the world, thereby stifling mental health (Ellis, 1974).

The cognitive turn in psychology has used this view and created robust frameworks which are vital for the mental health of individuals (Butler et al., 2006). Under such frameworks disturbed emotions and mental states have been swiftly corrected by cutting into the illogical and distorted thinking patterns of an individual. While such an approach is necessary it loses salience as higher dimensions of psychological health, such as having a deeper appreciation of beauty or spontaneity, are aspired for Assagioli (1989). In other words, psychotherapy based on scientific and cognitive models of mental health require complementary frameworks that can support deeper needs of the human consciousness. When these needs are addressed, the subtle dimensions of human health are opened up as illustrated by the following excerpt from Jane Austen’s famous novel Persuasion:

“…Mrs. Smith’s enjoyments were not spoiled by this improvement of income, with some improvement of health, and the acquisition of such friends to be often with, for her cheerfulness and mental alacrity did not fail her; and while these prime supplies of good remained, she might have bid defiance even to greater accessions of worldly prosperity. She might have been absolutely rich and perfectly healthy, and yet be happy. Her spring of felicity was in the glow of her spirits, as her friend Anne’s was in the warmth of her heart…” (Austen, n.d.).

These dimensions of mental wellness lie outside the scope of mainstream psychotherapies which have to tailor themselves for clinical formats, scientific testing in order to fit into the economic system of medical expenses (Leonhardt, 2021). Thus, it has become difficult for individuals to acquire such spaces of human consciousness by using solid knowledge frameworks that are supported by robust clinical and research work. Instead, it has led to the proliferation of pseudo-spiritual approaches which may appear promising on the surface but in reality are spurious and toxic to mental health. Often these approaches feed on the criticism of mainstream frameworks to push in fads which are of limited therapeutic value and have the potential to cause great harm to mental health (Assagioli, 1989). As a consequence, the discourse around mental health has underrated the whole issue of deeper aspects of psyche and their role in healing. However, with widespread social conflict and deteriorating psychological health of large sections of the population, these deeper aspects can’t be overlooked for long (Leonhardt, 2021).

Paradoxically these deeper aspects can be easily accessed by individuals as they lie embedded in their cultural resources. However, the process to enliven them lies outside the scope of the current discourse of mental health. Indic healing traditions of Yoga, Ayurved and Vedanta have long given space to both the subtle and gross aspects of mental health. In the ensuing sections, the paper explores the lived experiences of these traditions to explore how this integration has been made possible. In this way, the paper argues that if the role of cultural resources in mental health is given more space, then it can become an important step in the attainment and maintenance of positive health. This study bases itself on the strong foundations of several healing traditions and their scholars, notably Existential psychotherapy, Yoga, Ayurveda, and Vedanta.

## Mental Health

There are numerous ways of looking at mental health that range from purely biological approaches on the one hand to profoundly philosophical approaches on the other. The medical perspective gives more importance to neurobiological antecedents for mental health issues. However, unlike issues wherein other organs of the body are involved, mental health problems can occur both due to neurobiological as well as psychological causes. Often both causes tend to get mixed up, making mental health issues more problematic to address. Therefore, it is no surprise to find that the etiological factors of various mental health issues have not been clearly identified. In such a scenario, a comprehensive approach requires a wide spectrum of healing protocols to provide comfort and support to an individual. As a recognition of this current treatment guidelines use a combination of both pharmacotherapy and psychotherapy (Generalized Anxiety Disorder, 2021).

This article explores a third dimension of mental health which also needs to be made a part of the healing equation. This dimension looks at the nature of life itself and examines the possibilities of healthy mental states in it. In other words, this dimension explores the proposition that given the various constraints, influences and complexities of life, meaning of mental health cannot be restricted to biological and psychological aspects only (Schneider, 2019). It is imperative that for robust mental health the “givens” of life need to be discovered, accepted and negotiated. While the biological and psychological aspects may bring an individual to a firm ground and reduce the scope of negative emotions like dissatisfaction, irritation, frustration, anxiety, etc. they are unable to provide a sense of satisfaction, meaning and fulfillment to the individual by themselves.

In the absence of satisfaction, closure, meaning individuals are likely to become disillusioned and act in ways that may lead to psychological or biological problems. This in turn can disturb the mental health of an individual and it often becomes difficult to ascertain the triggers of the current poor state of an individual. This is so because the three dimensions of mental health namely biological, psychological and spiritual/existential are in a dynamic state of balance and changes in one aspect have consequent changes in the other. Hence, a comprehensive view of mental health needs to give due importance to existential issues as well (Yalom, 2020).

Existential Humanistic approach views health from a unique perspective viz. the ‘givens’ perspective. The ‘givens’ perspective is a deceptively simple concept, which is easy to understand but difficult to apply. This perspective points out that life as such has a challenging course wherein an individual encounters various existential challenges and her response to these challenges has a great bearing on her experience of life. Outside the existential framework, it has sometimes been looked down upon by other approaches as a fatalistic philosophy, one that is morose and boring. The Existential approach has often been misunderstood as pessimistic or impractical and is categorized as a philosophical approach with limited medical value. Indeed, the existential framework is a far more wide and profound framework which critiques mainstream mental approaches for their propensity to pathologize individuals and box them into categories.

A three dimensional perspective of mental health makes it evident that while issues might start in any one of the three dimensions, but the other dimensions are soon effected. In the initial stages, the other dimensions tend to act as a support system and help restore balance in the disturbed dimension. However, if the disturbed dimension remains in a state of problem then the other dimensions also start getting distorted. Thus, the dynamic equilibrium of the three major dimensions and their interconnectivity should be at the heart of a mental health discussion. A broad and unified view allows for early diagnosis and greater relief from pain and suffering for individuals.

For instance, let’s take the case of an individual who is biologically healthy. As there are no biological deficits in the individual, his mental health issues are likely to be less chronic and generally episodic. If the psychological factors are addressed by brief psychotherapy or psychoeducation then the individual can be brought to an even level wherein he feels disturbed emotions to a lesser degree or within normal limits. At this point the current mainstream mental health discourse tapers off and it is felt that unless some major challenges occur in the life of the individual he is likely to have a healthy experience of life. However, when individuals acquire stable mental health, their focus shifts toward acquiring deeper levels of satisfaction, peace and actualization of their potential. This forms the starting point of the existential dimension which dwells on the fascinating situation of a meaning seeking organism searching for meaning in a meaningless world (Frankl, 1985).

There can be broadly three outcomes of such a search for meaning by an individual. The first outcome being that of regression, i.e., faced with such a powerful existential question, the person finds himself over awed. Consequently, he leaves the quest for answering the deeper questions that bother him and returns back to the comforts of living with the knowns. In such a case, he may continue to live in a state of neither peace nor war scenario wherein an undercurrent of unease may be felt because he wants to express himself and realize his potential. To soothe and paper over this disquiet, the individual may also show an increased tendency for thrill seeking behavior as an attempt to shut down his higher sensibilities. This situation of existential limbo can continue for a long time, wherein there may be occasional instances of irritability, frustration and general displeasure. However, if a major challenge occurs in the life of such an individual then she may find it difficult to cope with it because her psychological reserves and resilience have been compromised due to her continual repressive efforts. With passage of time even small testing situations may become difficult to handle.

In the second outcome the individual may show fortitude and courage instead of getting overawed. Consequently, he starts to engage with his existential questions and makes efforts to seek guidance for handing his existential situation. Since the mainstream discussion on mental health considers such inquiries outside the scope of the mental health framework, the individual is unlikely to find much support. On the contrary, there is a greater likelihood of him getting confused and perturbed, prompting him to reconsider his attempts for deeper peace and wellness. These outcomes are encouraged by the vacuum which gets created by a reductionist view of mental health. Due to this vacuum, individuals tend to get attracted to pseudo-existential approaches that are usually filled with mystical, semi religious, dogmatic self-help fads that stand on very weak grounds.

There is little body of clinical work or philosophical basis for such fads and generally they are a collection of popular symbols, myths, and stereotypes about spiritualism. In the initial stages the individuals may feel he is making rapid progress in his quest for deeper meaning, but the hollowness of these systems soon catches up, and characteristically the individual opts out of these manipulative, exploitative systems as a disillusioned and dejected person. Herein, as in the first outcome, life challenges prove to be too overbearing for such an individual as he has limited reserves of psychological strength due to an unfulfilled quest for seeking maturity and meaning.

The third outcome could be of an individual who attempts to explore his existential moorings and is lucky to tap into reserves which equip him for such a journey. In such cases the individual is able to access support systems that are rooted in well researched, long traditions of intellectual inquiry into the nature of being. Such existential support provides the individual with the basic tools which are required for an existential quest. Equipped with these tools, the existential process becomes a gradual process of self-discovery and developing wisdom to understand the “givens” of life such as death anxiety, loneliness, responsibility and meaning. This kind of existential support may reach the individual in myriad ways. Sometimes it may be in the form of civilizational traditions such as poetry, art and spiritualism and at other times in the form of dialog, conversations and engagement with mentors or therapists (Shukla, 2021). The moot point being that a basic framework becomes the starting point of existential work upon which a life-long process of self-discovery and understanding tends to get built.

Without such a grounding it becomes difficult for individuals to separate the genuine from the spurious, especially in the face of sophisticated marketing campaigns which claim to provide wisdom and sense of purpose as a series of simple steps with a money back guarantee. Thus, the pathways that allow individuals to develop a basic grounding about the existential dimension are significant, as they enable individuals to make better choices about the resources they would like to tap into in their quest for higher dimensions of mental health.

## Indian Healing Traditions

Indian healing traditions give prime importance to mental health in the overall health and wellbeing of an individual (Shukla, 2020). For example, as per Indian traditions, the self exists at three levels, namely ‘Sthul Sharir’ or physical body, ‘ Shukshma Sharir’ or subtle body and ‘Karana Sharir’ or ‘astral/causal/existential body’(Halpern, 2017, August 11). These three bodies are different manifestations of the same being, and a condition of ill health affects the self at all three levels. Thus, it is said that “Adhi ke mool mein vyadhi aur vyadhi ke mool mein avidya’ meaning that behind the disease/discomfort in the physical body are incorrect cognitions that are present in the psychological self, which themselves arise out of lack of knowledge existing in the spiritual self). Therefore, in order to heal the self it is desirable to work on all the three bodies in a composite way since the three bodies or levels of existence are inter connected.

The Ashtanga yoga of Patanjali prescribes an 8 stage model of growth and rejuvenation namely yam, niyam, asan, Pranayam, Pratyahara, Dharna, Dhyan, Samadhi to work on the different dimensions of the self (Bryant, 2015). These different stages focus on specific pathways to reach the various levels of existence and include physical exercise, breath work, mediation, behavioral work and developing existential understanding. However, it is common to find that in spite of the huge popularity of Yoga as a means of health and wellness only the physical aspects of Yoga are given importance. In the absence of these deeper elements, the process of healing is incomplete as it does not touch the different levels of the self. A reason for this is the lack of understanding is a poor appreciation about the role of cultural resources in a healing/growth processes. Only few yoga protocols have been able to preserve these deeper aspects such as the Kundalini Yoga protocols which have been developed by David Shannahoff-Khalsa as taught by Yogi Bhajan (Shannahoff-Khalsa et al., 2019).

To help an individual reach deeper levels of existence and develop existential understanding, Indic healing traditions had developed various cultural resources such as Indic festivals. In the present times, such cultural resources do not receive much attention and have receded into the background. The role of cultural resources can be understood by looking at the deeper layers of Indic festivals and their linkages with Indian healing traditions. In their modern form, Indic festivals have become commercialized and have lost some of their subtle aspects of healing. Today, the focus of these festivals has shifted to consumption and marketing of various kinds of products and services. Yet, if the deeper aspects of these festivals are explored it gets revealed that these festivals still have the potential to provide an opportunity for individuals to do existential work.

One of the illuminating concepts of Indic healing traditions for approaching existential work is described in a classical treatise by Adi Shankaracharya known as the concept of ‘Shat Sampatti’ or the ‘Six treasures’(Tejomayananda, 2000). The stance of referring to existential understanding as the six treasures is to nudge the individual to experience a feeling of “awe” and initiate a process of healing by shifting our consciousness to the importance of mental health in our lives. The different treasures of ‘Shat Sampatti’ are elaborated as follows:

• *Shama*, or the ability to be calm and keep a peace of mind
• *Dama*, or the ability to control the senses and, therefore, reactions to external stimuli
• *Uparati*, or renouncing anything that doesn’t fit your dharma (duty)
• *Titiksha*, or persevering through suffering
• *Shraddha*, or trusting and having faith in the path of Jnana yoga
• *Samadhana*, or total concentration and focus of the mind

It can be discerned that these six qualities are interdependent and form a healing gestalt which allows a person to participate in existential work. For instance, to do existential work one must have trust and faith about the importance of existential understanding which can be seen from the aspect of *Shraddha.* Once such a trust is developed, a process of self-discovery begins which involves reflection, study and dialog. This aspect is referred to as *Uparati* wherein one is focused on the task at hand and is able to steer away from distractions. However, to stay steadfast on one’s journey in the presence of distraction requires self-control at the level of the mind and body. Shama & Dama, stand for these sensibilities and reinforce the fact that as one progresses on the journey of self-exploration, an individual shall have to negotiate with different life situations. A person who has resilience shall be able to successfully negotiate with the different kinds of situations and not go off track. Together with these psychological endowments a seeker needs to cultivate a sense of focus which is reflected by the aspect of *Samadhana.*

On a cursory look it may look that various dimensions of Shat Samapati are self-evident and are applicable to any important project in life. However, a deeper reflection will reveal that these psychological endowments are requisite for commencing on a journey of existential understanding. This leads us to the important question as to how these psychological endowments can be developed. Existential traditions point out that the role of dialog, study and reflection can be critical in the development of these psychological endowments. Yet, although the process of dialog, study and reflection may form the core of the process for developing existential understanding, the role of adjunct factors may be useful. It is here that Indic festivals due to their unique rituals, festivity and celebration create occasions wherein these abilities can be harnessed and kindled. At times these occasions provide an opportunity for advanced seekers to revise various aspects of existential wisdom, while at other times they start a process of self-discovery for young seekers. In the ensuing section the paper explores the various dimensions of Indic festivals and delves into their experiential side.

## Indic Festivals

Festivals are occasions of celebration in which people enjoy along with each other in a spirit of togetherness. They are an integral part of a culture and tend to develop over a long period of time. Indic festivals are no different and have a long history that goes back to the glorious traditions of Vedas and Yoga. Indic festivals are multidimensional and are rooted in the agricultural practices of the region. Thus, major Indic festivals coincide with the season of harvest wherein there is abundance of grain and agricultural produce. As a ripe crop is being harvested, it is also the time for sowing of a new crop. The time between harvest and sowing season becomes a time to reflect, relax and rejuvenate. Set in this background, the festivals become an occasion for positive affect and mental peace. With grains being in abundance, rich food and a lavish cuisine is strongly associated with Indic festivals, adding to the overall positive mood and joy of these times.

Traditionally, harvesting or sowing of crops required different sections of the society to cooperate with each other so that cutting, transport and storage of the crop can be properly done. All these activities were labor intensive and required the involvement of all the members of a household. The rituals of Indic festivals developed from these requirements and promoted prosocial behavior. Although Indian society has modernized, the timing, spirit and ethos of the festivals have remained the same. Even though the techniques of farming have undergone a significant change, the crop cycles of the region have remained steady. These crop cycles tend to coincide with seasonal changes like from winters to summers or from summers to winters. Traditional healing traditions in India like Ayurveda emphasize a balance between Purusha and Prakriti or the self and cosmos and therefore recommend that periods of seasonal change should result in corresponding changes in meals, daily routine, clothing and recreation to maintain a balance which forms the basis of health and wellness (Shukla, 2020). Health and wellness is described in these terms by the famous Ayurveda surgeon Sushrata, “Samadosha, samagnischa samadhatumala kriyaha prasanna atmenindriya manaha swasthya ityabhidheeyate.” which means “. that the doshas must be in equilibrium, the digestive fire must be in a balanced state and the tissues (dhatus) and malas (wastes) must work in a normal state. The sensor, motor organs and mind, atma must also be in a pleasant state. Such a person is called a healthy person or Swastha” (Mishra, 1997).

In order to help individuals to make changes in their daily routines, during times of seasonal change, the changes in lifestyle were formalized as oral traditions and rituals of the festivals. This enabled the knowledge regarding these changes/adjustments to be preserved and recorded. Indic traditions have a sense of unity among different knowledge frameworks and regarded festivals as occasions wherein wellbeing and harmony can be catalyzed through various means. So just as traditions of Ayurveda blended in with the iconography of Indic festivals so also were the features of yoga enmeshed in them to make them occasions of holistic rejuvenation.

Religious and divine aspects helped in giving a stable structure to the festivals such as allowing in fixing the dates in advance. They also helped in making these occasions spiritual by taking a stance of being careful with the consumption of material goods or hedonism. The festivals celebrate the universal values of health and well-being through an embodiment in the form of a deity. The worship of a deity through various rituals allows for merging of different cultural modalities and becomes a distinguishing feature of Indic festivals. The parapsychological, theological aspects of the festivals are beyond the scope of this article yet the lived experience of performing all the religious ceremonies can have a profound existential impact. The ensuing section discusses the various pathways through which Indic festivals catalyze a process of healing and rejuvenation. Following (Table 1) are a few of the most famous and widely celebrated festivals in India.

**TABLE 1:** Prominent Indic festivals and their characteristic features.

	*Diwali: the grand festival of lights*	*Holi: the vibrant festival of colors*	*Krishna janmashtami: the birth of lord krishna*
***Description***	During this festival of lights, houses are decorated with clay lamps, candles, and Ashok leaves. People wear new clothes, participate in family puja (worship), burst crackers, share sweets with friends, families and neighbors. It is one of the most popular festivals in India.	On the eve of Holi, people make huge (Holika) bonfires and sing/dance around it. On the day of Holi, people gather in open areas and apply dry/wet colors of multiple hues to each other.	People fast throughout the day and break it with a special meal after dusk. They visit temples, pray, dance, and sing bhajans (hymns) at midnight as a part of the celebrations of the birth of Lord Krishna. Often, small children dress up like Lord Krishna on this day. Images and picturization of Lord Krishna’s life story are depicted as tableau or “jhankis.”
***Significance***	The festival marks the return of Lord Rama, along with his wife Sita and brother Lakshmana, after a long exile of 14 years.	It signifies the victory of good (Prince Prahlad) over evil (Holika) and the arrival of spring.	It is the annual celebration of the birthday of Lord Krishna.
***Key attractions***	Homes decorated with fancy lights, candles and clay lamps, bustling shops and markets, fireworks and crackers.	Holika bonfire, playing with colors, and thandai (flavored milk with nuts & spices).	Janmashtami puja and festivities in the temples and jhankis of Lord Krishna
***When***	The darkest new moon night of Kartik month of the Hindu lunisolar calendar, which corresponds to mid-October – mid-November as per the Gregorian Calendar.	Full moon (Purnima) of the Phalgun month of the Hindu lunisolar calendar, which corresponds to the month of March of the Gregorian calendar.	8th day (Ashtami) of Krishna Paksha (dark fortnight) of the month of Bhadrapada according to the Hindu lunisolar calendar, which corresponds to August or September of the Gregorian calendar.
***Things to do***	Light diyas, decorate your home, share sweets and gifts with family and loved ones.	Holika bonfires and sing/dance around it, play with colors, eat sweets esp. Gujiya.	Visit Krishna temples and attend a special puja that includes bhajans and jhanki.

## Discussion

As described earlier, one of the distinguishing features of Indic festivals is the worship of a deity who is closely associated with the festival through religious stories. At a spiritual level the deity is an embodiment of some cherished universal values such as knowledge, wisdom, equanimity, dignity and prosperity. The whole persona of the deity, i.e., their form, symbolism, traits portray these values and are enlivened through stories and folklore. The process of worship such as recitals of sacred hymns and chants create a divine experience. This along with the celebratory mood of festivals make it easy to get closely connected to with these values as they get manifested in symbolic ways in various forms. Thus, one gets a chance to closely experience these values which may be akin to intense identity or actualization experiences. In humanistic existential traditions, such experiences have been referred to as peak experiences and can be moments of epiphany or satori (Maslow, 2013). In this sense they have the potential to provide enlightening insight which may lead to growth and rejuvenation. The main pathways through which a process of healing and growth may proceed can be delineated as follows:

### Experience of Awe

The divine/spiritual experience helps in developing an attitude of trust toward the universal values of health and wellness. In the routine of life these values appear as sterile, bookish and akin to verbal games. It becomes difficult for individuals to relate with them and develop a deep understanding about them. Thus, when these values are exhibited and enlivened by the rituals and iconography of the festivities they are brought to life. Such lived experiences results in moments of awe that have a transcendental quality and help us break out from our scripted patterns of behavior (Schneider, 2004). The experience of awe is enhanced due to the special situation formed out of a general mood of festivity and the addition of aesthetic elements emerging out of a process of worship. These experiences can result in curiosity about deeper aspects of self and mark the beginning of an existential/spiritual quest.

Experience of awe allows us to re-access those capacities which have become dormant due to fixation with competitiveness, peer pressure and the obsession of winning. As Rank put it “First comes the perception of difference from others as a consequence of becoming conscious of self… then interpretation of this difference as inferiority.” Rank (cited in Kramer, 1996). When we regather the aesthetic sense, the capacity of awe even our inferiority looks beautiful and evokes a sense of wonder and embrace. From this solid grounding it becomes possible for the individual to dig deep into the meaning of inferiority and ultimately transcend it. “The mere fact of difference,” according to Rank, “in other words, the existence of our own will as opposite, unlike, is the basis for the [self-] condemnation which manifests itself as inferiority or guilt-feeling” Rank (cited in Kramer, 1996). To handle such an enormous anxiety requires an art and aesthetic sense that gets rekindled by an experience of awe. The moments of relish within a festive atmosphere reintroduce individuals to an aesthetic sense which allows them to deal with their existential unconscious at a deeper level, and the same is exhibited in more enriching lives at an outer level.

### Insight About Symbols

The rituals performed during the Indic festivals involve a feeling of respect and reverence to the deity by offering him various items such as food, clothes and gifts as one would to a real person. The offerings are symbolic in nature and serve as a mark of affection to the universal values which are being manifested in the form of the deity in the here and now. Thus, working with symbols as one would in dream work in the celebrated traditions of existential psychotherapy an individual learns the importance of symbols and their role in psychological growth. This may lead to important learnings for the individual and enables him to apply this wisdom in his own life. For instance, a high need for achievement may make an individual very active leading to many accomplishments in life.

Yet, even after acquiring these accomplishments sometimes such individuals often feel a sense of incompleteness which goes contrary to their expectations. Although, they had thought that by achieving their goals they would find an enduring sense of satisfaction, instead even after the long struggle of completing their targets the hollowness of the situation disturbs them. Rollo May in his book, Man’s search for himself, captures it like this:

“…By that I mean not only that many people do not know what they want; they often do not have a clear idea of what they feel. When they talk about lack of autonomy, or lament their inability to make decisions – difficulties which are present in all decades- it soon becomes evident that their underlying problem is that they have no definite experience of their own desires or wants. Thus they feel swayed this way and that, with painful feelings of powerlessness, because they feel vacuous, empty…” (May, 2009, pg. 4).

When faced with such challenges individual surmise that since their accomplishments were not sufficient, they need to pursue new goals and set bigger targets. This leads to a formation of a vicious circle wherein compulsive striving followed by incompleteness occur recursively. Often, even when individuals are able to become high achievers in life by such compulsive striving their inner life remains highly impoverished due to loss of humanness.

Sometimes, individuals do realize that their approach to life is leading to diminishing returns but they are unable to break out of this vicious pattern of behavior. Herein, an insight about the role of symbols in life may come in very handy. It can start a process of inner working which can lead to the identification of those symbolic closings which the individual was yearning for but was not aware of due to his obsession with outer goals and targets. Such symbolic work can result in actualization of self and help a person to move forward in his journey of self-realization and psycho-synthesis.

### Working With Defense Mechanisms

Defense mechanisms prevent rational, well adjusted, existential information processing from taking place on a regular basis. This leads to the formation of unfinished gestalts that seek closure and sap the psychological reserves of a person (Perls et al., 1951). Often, these unfinished gestalts manifest themselves in the form of projection and reaction formation. The unfinished gestalts may get resolved when the conscious self gets engaged in some activity and other parts of the self can work on this incomplete gestalt leading to better adjustment and closure. However, the general routine of life does not provide us with opportunities wherein a balance between our conscious and unconscious selfs can be created. Festivals provide individuals with these occasions wherein the usual defense mechanisms are bypassed because of the general ambiance of celebration and festivity. This allows for readjustment of self and its conscious and unconscious parts without the hindrances which are caused due to defense mechanisms.

Many psychotherapeutic modalities that do not emphasize the centrality of a dialog also use this principle to initiate a process of healing. For instance therapies like eye movement desensitization and reprocessing (EMRD) use alternative bio-mechanical stimulation/activity during a therapy session to engage the conscious self so that the unconscious self can readjust itself (Oren and Solomon, 2012). This allows processing of embodied cognition or unprocessed psychological material which leads to the formation of different kinds of dysfunctional psychological symptoms in a client to be processed or assimilated. After assimilation clients witness lessening of dysfunctional symptoms and a better quality of life.

Indic festivals create a similar lived experiences wherein the aesthetic settings allow individuals to get absorbed in the here and now leading to processing of unfinished gestalts. Once individuals witness such therapeutic effects they may become aware about new ways to address their personal issues. At the same time individuals gain insight about using different kinds of cultural resources for re-setting or rejuvenating their personality systems. In this way cultural resources help in preventing deterioration of sub clinical symptoms into full fledged pathologies.

### Invitation to Be in the Here and Now

Present Centeredness is an important aspect of mental health in humanistic traditions (Perls et al., 1951). The ability to stay in the here and now is both a sign of health and a therapeutic process in itself. With wide spread awareness about mindfulness it has become common knowledge that staying in the present moment is an important pathway for a richer life. Yet, even after such awareness many individuals find it very difficult to practice mindfulness and conclude it to be impractical. On the other hand, there is another category of individuals who after knowing the utility of mindfulness try to achieve it in such a mechanical way that the soul of the process is itself lost. Such individuals try to explore so much about the various techniques of mindfulness that they get lost and confused about which technique is to be followed. Paradoxically, it is this very fussiness that mindfulness can help an individual transcend. Yet, by bringing in a hyper achievement oriented mindset, a simple process of being in the moment is converted into a complicated affair, wherein a whole gamut of software and gadgets are used in order to track/monitor mindfulness.

An important aspect which supports such a dysfunctional approach is the discourse of hyper competitiveness which is promoted by a consumer oriented, consumption driven socioeconomic system. With this being the dominant discourse, individuals are fixated with its processes and tend to use it in those areas of life which require a different approach. Thus, even when trying to be mindful, the strong desire of intellectualizing and quantifying it is difficult to curtail. As Kierkegaard writes in The Sickness Unto Death,

”A self is the last thing the world cares about and the most dangerous thing of all for a person to show signs of having. The greatest hazard of all, losing the self, can occur very quietly in the world, as if it were nothing at all. No other loss can occur so quietly; any other loss – an arm, a leg, five dollars, a wife, etc. – is sure to be noticed”(The Guardian, 2010).

In such cases, if the dominant discourse is set in abeyance for sometime, then individuals may find it easy to be present centered. Indic festivals create such an atmosphere by nudging people to be prosocial, collaborative and accommodating of each other. The celebratory aspects, rituals, worship and customs lead to easing of competitiveness and individuals can come in the here and now. Such occasions can lead to insights wherein individuals may reassess their approach toward life and understand that they need to create spaces wherein this dominant discourse of competitiveness is not intrusive. When mindfulness practices unfold with such an understanding in the background, it becomes easy to integrate them as a part of self-leading to lasting growth and healing.

The above pathways highlight how Indic festivals create an atmosphere of healing and rejuvenation. The positive affect created by a mood of celebration, togetherness and joy becomes the cornerstone for exploring the subtle aspects of health and wellness. It is easy to be accommodating, adjusting and forgiving when the self is in such a state of elevated mood. At a subtle level these existential aspects get integrated with the physiological elements such as changes in lifestyle as per the traditions of Ayurveda and also behavioral dimensions of prosocial behavior. In this way, Indic festivals operationalize the Indian philosophical thought, that advocate an integrated approach of wellness wherein all the three levels of self are touched upon in a process of healing.

However to reach the deeper layers of self through the experience of festivals requires certain competencies to be present in an individual. These competencies have been identified in the Indian tradition as the Shat Sampatti or intellectual wealth. By identifying them as wealth the focus is on the fact that just as wealth allows us to survive and transact in this world, in the same way intellectual wealth is necessary to carry out the business of life. Shat Sampati or Intellectual wealth allows us to study, reflect and meditate about the deeper aspects of life. With such competencies in the backdrop, Indic festivals become occasions wherein individuals can experience awe, symbols, spontaneity and present centeredness.

It is pertinent to note that this paper has described Indic festivals in a purer sense wherein they are celebrated in an enlightened way. However, in their present form it can be argued that they are celebrated in a rigid, structured, overly commercialized manner which pushes the healing elements into the background. The purpose of this paper was to show that these are healing elements which are present in the backdrop and can still be accessed provided these exist a basic understanding of existential wisdom. When celebrated from this sensibility, Indic festivals have the potential to be relaxing, rejuvenating and recuperating.

## Conclusion

It is evident that redefining the contours of the present discourse of mental health allows us to access those resources which otherwise lay untapped. Not only do these resources give individuals more options in their quest for positive mental health but they also provide some unique benefits which are not available from the mainstream methods. The importance of these resources is further enhanced by the role they play in supporting the gains which are made by pursuing the mainstream modalities. These aspects need to be given due recognition and attempts should be made to incorporate them in the pursuit of mental health and wellness.

One of the ways in which these resources can be tapped is by accessing the sources of existential wisdom that lie embedded in culture. Usually, these resources are dormant and remain inaccessible because a basic understanding about the importance of existential aspects and their importance is not appreciated. This paper through the lived experience of Indic festivals shows the important role which existential wisdom can play in creating a solid foundation for mental health and wellbeing. It also highlights that once a basic grounding about the existential givens is developed, the individuals themselves become capable of accessing the existential wisdom which lies embedded in the culture of which they are a part of or have a fondness for.

Since such a search for existential wisdom is the journey of a lifetime and emerges from the lived experience of a person it is difficult to map it exhaustively. This may be regarded as a limitation of this study and future studies may try to address this gap. However, this paper argues that existential issues require a different kind of sensibility and therefore methodologies of addressing them should be different. For instance, developing a spirit for celebration and rekindling the ability to enjoy has been touched upon in the discussion on Indic festivals. It is important to note that clients who are diagnosed with Major Depressive Disorder show a loss of the ability to enjoy. If treatment proceeds primarily at a biological level through the use of antidepressants, then it may be not as effective and therefore current guidelines augment it with cognitive behavior therapy/interpersonal therapy (Major Depression, n.d.). This paper tries to show that if the existential aspects are also given some space in the treatment modalities then better results may be possible in the treatment of such disorders. However, unlike other modalities existential attempts are like a work of art wherein one proceeds with a sense of relish which gets disappeared when approached with a lot of focus on process and regimentation. They are best proceeded with a good understanding and sense of calm which is perhaps best captured by Viktor Frankl as follows:

“…Don’t aim at success. The more you aim at it and make it a target, the more you are going to miss it. For success, like happiness, cannot be pursued; it must ensue, and it only does so as the unintended side effect of one’s personal dedication to a cause greater than oneself or as the by-product of one’s surrender to a person other than oneself. Happiness must happen, and the same holds for success: you have to let it happen by not caring about it. I want you to listen to what your conscience commands you to do and go on to carry it out to the best of your knowledge. Then you will live to see that in the long-run—in the long-run, I say!—success will follow you precisely because you had forgotten to think about it…” (Frankl, 1985, pg. 16).

## Author Contributions

The author confirms being the sole contributor of this work and has approved it for publication.

## Conflict of Interest

The author declares that the research was conducted in the absence of any commercial or financial relationships that could be construed as a potential conflict of interest.

## Publisher’s Note

All claims expressed in this article are solely those of the authors and do not necessarily represent those of their affiliated organizations, or those of the publisher, the editors and the reviewers. Any product that may be evaluated in this article, or claim that may be made by its manufacturer, is not guaranteed or endorsed by the publisher.
